# A qualitative investigation of the perceptions of complementary and alternative medicine among adults in Hawaiʻi

**DOI:** 10.1186/s12906-022-03603-3

**Published:** 2022-05-07

**Authors:** Brittany R. Odegard, Mollie R. Ferguson, Farah Naja, Jennifer Ayoub, Jinan Banna

**Affiliations:** 1 Department of Human Nutrition, Food and Animal Sciences, College of Tropical Agriculture and Human Resources, Agricultural Sciences 314C, University of Hawaiʻi at Mānoa, 1955 East-West Rd, Honolulu, HI 96822 USA; 2 Department of Nutrition and Food Sciences, American University of Beirut, PO Box: 11-0236, Riad El Solh, Beirut, 1107 2020 Lebanon

**Keywords:** Hawaiʻi, Interviews, Qualitative research, Perceptions, Complementary and alternative medicine

## Abstract

**Background:**

Complementary and alternative medicine (CAM) is defined as a group of diverse medical and healthcare practices outside of conventional medicine modalities. The use of CAM is steadily increasing despite gaps in the scientific evidence supporting its use and the challenges of its regulation and integration into conventional healthcare practices. In this context, perceptions concerning CAM become important. The purpose of this study is to identify the perceptions of CAM among adult residents of Hawaiʻi.

**Methods:**

Two researchers conducted audio-recorded interviews at the University of Hawaiʻi Mānoa (UHM) campus. Participants were over the age of 18, spoke English fluently, and self-identified as Hawaiʻi residents. Interviews were conducted to the point of data saturation and audio recordings were transcribed verbatim. Researchers collaboratively developed a codebook and used NVivo 12 to analyze transcripts. New codes were added as required. Inter-rater reliability was determined by calculating Cohen’s kappa coefficient. Key themes were identified by both researchers individually and then discussed and evaluated together.

**Results:**

Participants were mostly female, white, and affiliated with UHM. Perceptions were categorized as positive, negative, and neutral. The majority of participants had positive perceptions of CAM with few reporting negative perceptions. The positive perceptions were related to CAM’s perceived effectiveness, the desirability of CAM compared to conventional medicine, and CAM’s ability to foster well-being. Negative perceptions were attributed to the lack of scientific evidence and ineffective outcomes of CAM use. Neutral perceptions of CAM related to its safety and natural characteristics.

**Conclusions:**

The study found mainly positive perceptions of CAM among Hawaiʻi residents. Use of CAM is on the rise despite CAM lacking robust empirical evidence demonstrating efficacy across various medical conditions. With insufficient data and understanding of current medical literature, CAM users place themselves at risk for harmful herb-herb and herb-drug interactions. These findings have implications for healthcare providers of both conventional medicine and CAM traditions.

**Supplementary Information:**

The online version contains supplementary material available at 10.1186/s12906-022-03603-3.

## Background

The National Institute of Health’s Center of Complementary and Integrative Health (NCCIH) is a US federal agency dedicated to complementary and alternative medicine (CAM) research. The NCCIH defines CAM to be a group of diverse medical and healthcare practices and products that are not presently part of conventional medicine [1]. Complementary medicine refers to non-mainstream practices being used together with conventional medicine. Alternative medicine refers to non-mainstream practices being used in place of conventional medicine. There are many types of complementary and alternative approaches. These include application of natural products such as herbs, vitamins, minerals, and probiotics. In addition, there are mind and body practices such as yoga, chiropractic, meditation, acupuncture, and tai chi. Other CAM therapies include the practices of traditional Chinese medicine, Ayurvedic medicine, homeopathy, and naturopathy. Before existence of modern medicine, it is inferred that CAM practices have been used in diverse cultural populations throughout history as primary medical care.

Evidence suggests that CAM use is increasing globally [2]. The increasing awareness surrounding CAM use is highlighted by the 2019 World Health Organization (WHO) global report on CAM that shows an increase of member states implementing national policies and developing national laws and regulations [3]. Furthermore, the WHO is concerned about the safety of users due to the lack of policy regulations, scientific evidence, and support of integrating CAM into the healthcare systems [2]. In addition, previous studies show that the use of CAM may not be universally safe due to the high risk of harmful herb-herb interactions, herb-drug interactions, and direct herb toxicity [4].

Over the last 20 years, there has been a plethora of published literature in the field in response to CAM’s popularity [5]. The first national CAM survey completed in the United States (US) was in 1990. It reported that 34% of adults had used CAM at least once in the past year [6]. Seven years later, a follow-up survey reported an increase of CAM use to 42% [7]. In 2002, a separate report found that 62% of American adults received provider-delivered CAM [8]. The most common types of CAM used in the US involve use of natural products, and the practices of yoga and meditation [9]..

In 2007, a quantitative research study was conducted using data from the Hawai’i Health Survey (HHS), which represented all of the Hawaiian Islands. This research revealed that the use of provider delivered CAM is more popular in Hawaiʻi compared to the US mainland [10]. Hawaiʻi is a small island chain state located in the Pacific Ocean, situated between the west coast of the continental US and to the east of many Asian countries. Therefore, the influence of different cultures such as the Chinese, Japanese, and Native Hawaiian culture may contribute to this characteristic use of CAM in Hawaiʻi.

Previous research of CAM in Hawaiʻi was designed to understand the prevalence of CAM and the types of CAM that were used with residents who had cancer [11, 12]. There are no known qualitative studies related to CAM in Hawaiʻi. It is essential to determine what the public knows and believes about CAM to provide insight to policy makers and physicians so they may effectively regulate, educate, and implement safe patient care practices. Qualitative studies of CAM are important to gather meaning and understanding of CAM. For instance, a qualitative study among Australian women showed that the motives for CAM use fell into three main categories, the “push” factors repelling people from mainstream medicine, the “pull” factors attracting people to CAM use and the barriers to CAM use [13]. The push and pull factors have also emerged as determining factors of CAM use in other populations [14, 15]. Further studies may reveal the underpinnings of CAM use and explain why CAM is increasing in popularity despite a dearth of supporting scientific evidence [16–18]. The objective of this study is to identify the perceptions of CAM among adult residents of Hawaiʻi.

## Methods

This study was approved by the Institutional Review Board at the University of Hawaiʻi at Mānoa (UHM). All participants read and signed a consent form prior to participation in the study. Participants received a $10 gift card as an incentive following interview completion.

### Participants

Participants for the study were recruited using flyers posted at the UHM campus and local community boards, email list-serves, and social media posts. Those who were interested inquired by email and were sent a screening survey on Google Forms to determine eligibility. Eligible participants were 18 years old or older, self-identified as Hawaiʻi residents, and spoke English fluently. Twenty-one individuals participated in the study (*n* = 21).

### Interview guide

Two researchers (BO and MF) conducted audio-recorded semi-structured interviews using an interview guide composed of open-ended questions. The interviews were conducted with either one or both interviewers present. BO and MF asked follow-up questions when necessary. The guide facilitated dialogue and expression of personal beliefs, attitudes, and experiences. The guide was developed based on existing CAM literature and from an adapted version of the social-behavior model (SBM) [19–21]. The SBM is recognized for being a successful conceptual model to predict CAM use and used in many previous studies relating to CAM [22, 23]. The adapted version includes health beliefs known as *push factors* and *pull factors. Push factors* are the reasons that led to a dissatisfaction of conventional medicine, while *pull factors* are the reasons that related to the desire for a more holistic approach or having a more proactive approach [19].

The interview guide was pilot tested with three participants to ensure that participants understood the questions. Necessary modifications were made to the interview guide. Given that there were minimal modifications of the interview guide, data from the pilot interviews were used in the data analysis.

### Initial codebook development

A codebook was required to systematically categorize responses from the participants and assign meaning to the text. The preliminary codebook, including definitions of each code, was created by examining existing literature [19]. The codebook was organized by perceptions of CAM, types of CAM used, and motivators for use of CAM. For example, codes in the “perception” category included “satisfaction” and “safe”. Codes for the types of CAM included “acupuncture”, “meditation”, and “supplements”. Lastly, codes in the motivators for use included “dissatisfaction with conventional medicine” and “inexpensive”. The researchers (BO and MF) modified and expanded the codebook as interview transcripts were analyzed.

### Audio-recorded interviews

JB, who has extensive experience conducting qualitative research, provided both interviewers (BO and MF) with qualitative interview training. All interviews were held on the UHM campus in private rooms.

At the beginning of the interviews, the participants were asked questions such as, “When you hear the term CAM, what comes to mind?” and “What therapies do you associate with CAM?” The NCCIH definition of CAM was provided to further assist the participants during the interviews. The remaining questions were asked, such as, “Why do you use CAM?”, and “Have you discussed the CAM products/therapies you use with your doctor?” The average length of the interviews was approximately 30 min. Following each audio-recorded interview, the researchers and assistants transcribed the audio file verbatim into a word document. The two researchers (BO and MF) quality checked each completed transcription to ensure accuracy by listening to the audio and reading the transcripts.

### Data analysis and final codebook development

The two researchers (BO and MF) used the NVivo 12 qualitative data analysis software (QSR International Inc., Burlington, MA, USA) independently to perform directed content analysis of all the transcripts [22]. All data analysis of transcripts were completed independently and then discussed between BO and MF to reach an agreement for the final results.

Firstly, inter-rater reliability was evaluated between BO and MF. The two researchers analyzed and coded the first three interview transcripts using the initial codebook. Then, a calculated Cohen’s kappa coefficient of 0.66 was derived from the results in NVivo 12 and interpreted as acceptable agreement between the two coders [23]. After reaching agreement, BO and MF analyzed and coded an additional 10 transcripts. Next, BO and MF discussed the results from the first round of coding. BO and MF determined there was a need for new codes and organizational edits of the initial codebook. This discussion led to the creation of a second codebook with newly organized categories that described the different emerging perceptions of CAM: positive, negative, and neutral. These new categories were based on the highest code counts and themes derived from participant quotations. Some examples of perception codes were “effective”, “safe”, and “natural”. The second codebook is the final codebook and no new codes were added following this edition.

Secondly, BO and MF re-analyzed the first 13 transcripts using the final codebook. Data saturation was determined after coding these 13 transcripts, when BO and MF found no new emerging themes [24]. Despite reaching data saturation, BO and MF coded the additional eight transcripts collected to verify that there were no other codes needed to be added. A total of 21 transcripts were analyzed.

Lastly, BO and MF independently evaluated key themes and perceptions identified from examining code counts and quotations. Afterwards, the two researchers discussed and agreed on the final themes and collectively summarized the results.

## Results

Most participants were female (81%), identified as Caucasian (57%), and had completed some higher education. The characteristics of participants are displayed in Table 1. The CAM therapies participants most commonly used were vitamin and mineral supplements, herbs and botanicals, yoga, and meditation. See Table 2 for the frequency of all reported CAM use. Three participants identified as non-CAM users since they did not report use of CAM within the last 6 months prior to their interview. The remaining 19 participants identified as current CAM users. Ten of the 19 CAM users explained that they did not speak with their doctor about their CAM use.

**Table 1:** The socio-demographic characteristics of research participants (*n* = 21)

Variables	Total (n[%])
Age (yrs)	
Mean ± SD	31.2 ± 16
Gender	
Female	17 (81%)
Male	4 (19%)
Race/ethnicity^a^	
Asian	10 (47.6%)
Native Hawaiian	1 (4.8%)
Pacific Islander	1 (4.8%)
Alaska Native	0
American Indian	0
African American	1 (4.8%)
White	12 (57.1%)
Hispanic	3 (14.3%)
Not Hispanic	18 (85.7%)
Highest Education Level	
Primary Education	0
High School	2 (9.8%)
Trade School	0
Some College	10 (48.8%)
College Degree	8 (41.5%)
Marital Status	
Single	13 (61.9%)
Married	6 (28.6%)
Divorced	2 (9.5%)
Widowed	0
Re-married	0
Annual Income	
< $10 k	8 (38.1%)
$10 k-$20 k	4 (19%)
$20 k-$40 k	4 (19%)
$40 k-$60 k	1 (4.8%)
$60 k-$80 k	1 (4.8%)
$80 k-$100 k	0
> $100 k	3 (14.3%)
Employment Status^a^	
Employed	15 (71.4%)
Self-employed	2 (9.5%)
Unemployed	0
Homemaker	0
Student	13 (61.9%)
Retired	0
Unable to Work	0

^a^Check all that apply

*Legend*: This table summarizes the socio-demographic characteristics of this study’s research participants. The majority of the participants were white and female and made less than $10,000 per year

**Table 2:** The types of complementary and alternative medicine (CAM) used among participants (*n* = 21)

CAM Therapy	Total^a^ (n[%])
Vitamin and Mineral Supplements	12 (57%)
Herbs and Botanical Supplements	12 (57%)
Yoga	11 (52%)
Meditation	10 (48%)
Diet	8 (38%)
Traditional Chinese Medicine	7 (33%)
Acupuncture	6 (29%)
Massage Therapy	6 (29%)
Ayurveda	2 (10%)
Reiki	1 (5%)
Cryotherapy	1 (5%)

^a^Some participants used more than one CAM therapy

*Legend*: This table shows which types of CAM are used among research participants. Some participants used one therapy while others used more than one. The most common CAM therapies used were vitamin and mineral supplements, and equally herbs and botanical supplements. Yoga and meditation followed the most common. The least common therapies were Ayurveda, Reiki, and Cryotherapy

Participants had a range of perceptions that were organized as positive, negative, and neutral. However, there were more positive perceptions identified than neutral and negative perceptions of CAM.

### Positive perceptions of CAM

Positive perceptions were those that seemed to motivate the participant to use CAM. The three main positive perceptions were: 1) CAM is effective, 2) CAM is “better” than conventional medicine, and 3) CAM fosters well-being. In fact, 19 out of 21 participants had positive perceptions of CAM.

Additional file 1 presents the themes and associated exemplifying quotations for positive perceptions in form of a table [see Additional file 1].

1. CAM is effective (push factor)

Eight participants had the perception CAM is effective in treating their ailments. CAM therapies that were reported to improve symptoms include vitamin and mineral supplements, herbal supplements, massage therapy, and therapeutic diets. Many participants compared their trial-and-error with conventional medicine, stating “CAM was more effective than conventional medicine.” Some participants who used CAM mentioned ineffectiveness of certain practices or products. Notably, the reported ineffectiveness did not negatively impact overall positive perceptions of CAM due to effectiveness of other practices and products used.

2. CAM is better than conventional medicine (push factor)

Seven participants perceived that CAM is “better than using conventional medicine”. Participants with this belief supported CAM’s holistic approach and its ability to treat the root cause of disease. Participants also appreciated minimal side effects during CAM treatment.

3. CAM fosters well-being (pull factor)

Five participants had positive perceptions of CAM due to increased feelings of health and happiness following CAM use. In addition, two participants described their use of CAM outside of treating an ailment and used CAM for the purpose of “feeling good” or for “fun.”

### Negative perceptions of CAM

Negative perceptions were those that seemed to deter participants from using CAM. Three participants shared negative perceptions. Although few, the two main negative perceptions identified were: CAM is lacking scientific evidence/regulations and CAM is ineffective. See Table 3 for themes and associated exemplifying quotations for negative perceptions.

1. CAM is ineffective

**Table 3:** The participants’ negative perceptions of complementary and alternative medicine (CAM)

Negative Perception (n)	Definition	Exemplifying Quotations^a^
CAM lacks scientific evidence and regulations (3)	The perception that CAM does not have sufficient scientific evidence.	“I just didn’t feel like some of them [research articles] had the evidence”^a^ (P17)
		“There’s lots of things that have little or no evidence to support [CAM]. It’s just wishful thinking.”^a^ (P19)
		“If I add a supplement to a pig diet, it has to be tested to prove that it is safe and effective. Those standards do not apply to herbal products used in human medicine.” (P19)
CAM is ineffective (2)	The perception that CAM did not provide the participant desired results.	“I feel like CAM medicine therapies haven’t working for me so far, so I just stick to the western medicine.” (P16)
		“I was not satisfied because I had a negative reaction and really no improvement.” (P17)

^a^Quotations edited to add context and for grammatical purposes. P, participant number

*Legend*: This table highlights the negative perceptions identified from participants. Only 3 participants had negative perceptions. The two major concepts leading to negative perceptions were the lack of scientific evidence and regulations, and the ineffectiveness of CAM. Each concept has a definition to help code and organize the transcripts. The exemplifying quotations are the statements of participants that were identified to belong in the theme of negative perceptions

Two participants stated the CAM therapies they utilized were completely ineffective, which led to their dissatisfaction.

2. CAM lacks scientific evidence and regulations

Two participants expressed their distrust in CAM due to lack of scientific evidence. One participant reasoned there is *“sparse data and a lack of double blinded, randomized controlled trials”* and regulations for CAM are “*less strict for humans when compared to animals”.*

### Neutral perceptions of CAM

Neutral perceptions related to CAM are those which either describe CAM and its general characteristics or a perception having both positive and negative elements. There was two main neutral perception of CAM: CAM is safe occasionally and CAM is natural. Additional file 2 shows themes and associated exemplifying quotations for neutral perceptions in a table format [see Additional file 2].

1. CAM is safe occasionally

The perception that is CAM safe for occasional use was very common. Fourteen participants shared that CAM use safety depends on many factors such as: the reliability of where information is sourced, the CAM provider’s scope of education and knowledge, the technique and application of CAM therapies, the potential herb-herb or herb-drug interactions, the risk of overdose, and/or the user’s health status. This perception seemed to not motivate the CAM users.

2. CAM is natural (pull factor)

The perception that CAM is natural was common. Nine participants described CAM to be natural because “it comes from nature” or is “not made in a lab”**.**

## Discussion

This research study is the first known qualitative study in Hawaiʻi developed to identify the perceptions of CAM in adults. The past studies of CAM in Hawaiʻi were quantitative and provided data on the types and frequency of CAM that was used [10–12, 25]. Findings of the current study may have implications for local and national policy makers, medical and CAM practitioners, and future research studies.

It is important to note that the definition and terminology of CAM is evolving. It can be seen through the history of the NCCIH, which has undergone several name changes in the past in efforts to embody an accurate definition and label. There has been ongoing debate about how to define CAM and what constitutes CAM use [26].For instance, in other countries where CAM therapies are the primary method of care, the terms *complementary* and *alternative* do not apply [27]. For the purpose of this study, the NCCIH definition was provided to the participants during the interview to minimize ambiguity.

The perceptions of CAM may influence the choice to use or not use these therapies. The results of our study demonstrated the perceptions of CAM among participants were mainly positive. All 19 self-reported CAM users had positive perceptions while the three reported non-CAM users had negative perceptions. Our participants were predominantly female (*n* = 17). Of note, previous studies have demonstrated that females are more likely to use CAM [28–30]. However, one past study in Hawaii has reported that the use of provider delivered-CAM was comparable between males and females [10]..

The perception that CAM is effective is the most common positive perception in the current study and was found to be commonly observed in previous studies. A study of 13 menopausal women in Canada reported that the participants believed their personal CAM use was effective in the treatment of their symptoms [31]. Additionally, Bahall et al. similarly reported Trinidad cardiac patients believed CAM use improved their health and increased positive clinical outcomes [32]. Another study conducted in South Korea on 649 young adults also reported that more than half of the participants perceived CAM to be effective at relieving their pain and other symptoms [33]. In this context, we could suggest from our results that users of CAM are generally satisfied with its outcomes. Like in the studies mentioned, CAM accomplished and reached their expectations of the treatment, which is why the users have positive perceptions.

The perception that CAM is “better” than conventional medicine, and therefore more desirable, has also been reported in previous studies. It is perceived that CAM has minimal side effects compared to conventional medicine. Bahall et al. similarly reported that CAM use in Trinidad cardiac patients was greatly favored due to conventional medicine’s undesirable side effects [34]. This result was repeated in a study of breast cancer survivors, which revealed some participants decided to use CAM because conventional medicine was not effective and its side effects required additional medical treatment [35]. As participants in the current study reported, a major push factor for CAM use is dissatisfaction of conventional medicine, and specifically due to the undesirable side effects that come from conventional medicine treatment plans.

The perception that CAM fosters well-being can be seen in other studies. For example, a study of type 2 diabetic patients in Taiwan revealed participants utilize CAM to improve their well-being, but also perceived CAM to be a necessity in their treatment of type 2 diabetes [36]. In contrast, a research study in Southern England described a phenomena of CAM use as a “treat” rather than a “treatment” [37]. A “treat” was a personal luxury or desire in the absence of a health need. In contrast, a “treatment” was utilized as a mean of prevention, alleviation, or cure for specific health ailments [37]. Results from these two studies mirror the perspective of the majority of our participants who claim CAM helps them feel good when used as a treatment and/or as a treat to foster well-being.

In addition, neutral perceptions of CAM corresponded to the natural and holistic characteristics. There is a common belief among CAM users in which CAM treats the person in a holistic approach by solving the root cause of disease, making it a highly favored treatment strategy. Nichol et al. reported similar ideals after having conducted a qualitative study of 15 families in Bristol, England [38]. The results demonstrated a recurring perception that CAM is natural and holistic, meaning there is little to no manipulation by man in a lab. In contrast to the present study, the participants also reported an overall “negative definition of CAM” because they thought CAM was not “medicine”. Another study in menopausal women from California showed that most of its participants considered CAM to be natural, which was desirable [39]. Another neutral perception of CAM in our study is related to CAM’s safety, in that it is not universally safe and is dependent on variable circumstances.

The negative perceptions of CAM the current study found is comparable to a qualitative study conducted with head and neck cancer patients [40]. Similarly, this study had more positive perceptions with few negative perceptions. Of the negative perceptions, the participants shared that CAM is ineffective, is a mere placebo, and is unregulated. These results are similar to those of the current study.

This study’s results have implications for policy development, practice implementation, and future research. These findings could further promote efforts to integrate CAM into mainstream health care systems through policy development and augmentation for the inclusion of CAM education provisions for all health care providers (HCP) [41]. Our data revealed that 10 participants didn’t discuss their CAM use with their doctor. Evidence suggests patients have a tendency to withhold CAM use from their healthcare providers [42]. An Australian study showed 57.2% of 1270 CAM users did not report to their doctor [43]. Meanwhile, a study of 438 physicians in Italy revealed that about half of the participants did not engage their patients in discussions about CAM [44]. A 2015 US study of 1767 adult CAM users reported 46.7% did not inform their doctor about their CAM use [45]. In the current study, most CAM users identified as being a complementary user because of their dual practice of conventional medicine and alternative medicine. Thus, communication between the HCP and their patients becomes a critical element of providing safe and effective treatment plans. An example of the integration of CAM through policy implementation would be expanding the HCPs’ scope of practice to require CAM education for each patient and to encourage HCPs to engage and initiate conversations with patients about their CAM use.

CAM use in Hawaii may be influenced by the connection between ethnicity and which CAM remedy is utilized. Results from a national study in the United States concluded CAM use differs among groups and ethnicity plays an independent role in the use of CAM modalities, the health problems for which CAM is used, and the choice of CAM practitioners [46]. Another study in California further supports the notion that patterns of CAM use and ethnic-specific CAM use vary across racial/ethnic groups [47]. This study concluded evaluation of CAM use in ethnically diverse populations should recognize ethnic-specific modalities and variation across ethnicities, which continues to emphasize the deep need for HCPs to construct cultural appropriate screening of CAM use within their patient populations.

The current study’s results also have implications for the safety and well-being of patients. Due to positive perceptions, the patient may unknowingly harm themselves when using CAM due to lack of professional guidance and inaccurate knowledge. It is the HCP’s responsibility to be fully aware of all CAM and non-CAM therapies in which their patients partake, as there is a risk of drug-drug and drug-herb interactions. To elicit this information from patients, HCPs would benefit from taking a patient-centered approach to foster a welcoming and non-judgmental environment. Engaging in the practice of patient-centered care will ensure the patient communicates about their CAM use or desire to use CAM.

In addition to integration of CAM into patient-practitioner discussion, our results reveal the importance of promulgating CAM products, such as herbs, vitamins, and minerals. The most common types of CAM products used in the current study are vitamin, mineral, and herbal supplements. Results in previous studies related to Hawaiʻi CAM [10, 12] suggest that natural products are the most common types of CAM used. The lack of regulatory policies could have an impact on CAM users’ safety and the efficacy of CAM as a whole-person treatment system. For instance, risks of taking unregulated CAM supplements include possible vitamin/mineral toxicities and heavy metal exposure. Current FDA regulation regards dietary supplements as food rather than as drugs. The result is fewer and less strict FDA rules for dietary supplements compared to drug regulations [48]. FDA policy purports that dietary supplements are not making claims to diagnose, treat, cure, or prevent disease. The perceptions of CAM users indicate beliefs contrary to FDA policy.

Future research about CAM is warranted. First, additional rigorous studies to evaluate the efficacy of specific CAM treatment interventions should be conducted. Secondly, an in-depth analysis of conventional medicine and CAM integration should be conducted to explore common beneficial and protective factors, combination potentiating factors, and detrimental risk factors. The potential of how perceptions impact health care decisions, and how positive perceptions correlate with CAM use, should be explored. These results could inform CAM providers and guide educational efforts in medical schools. Additional studies should be conducted to investigate the effect of various ethnicities on CAM use and if utilizing at home CAM treatments are more cost effective. Lastly, the triangulation of perspectives between healthcare professionals not providing CAM, healthcare professionals providing CAM, and active CAM users is needed in order to gain deeper insight into the integration of CAM into the entirety of the prevailing healthcare system.

## Limitations

Most participants were affiliated with UHM. The data gathered in this study may not reflect views of the wider population of adults in Hawaiʻi. In addition, the study may have been subject to self-selection bias because participants may have volunteered to participate due to an interest in CAM. However, the goal of this qualitative study was to present a rich description of perceptions of CAM use rather than to derive results that may be generalized to a broader group. This study does not quantify the impact of perceptions and use of CAM on health; it presents only how participants felt about their CAM use, or lack thereof.

## Conclusion

This study revealed positive, negative, and neutral perceptions of CAM in adults. More positive perceptions were identified than neutral and negative perceptions. The most common positive perception was related to CAM’s effectiveness. The most common negative perception was that CAM is ineffective and lacks empirical scientific support. The most common neutral perception is CAM is safe. Overall, participants’ perceptions of CAM were positive given the perceived lack of side effects, effectiveness, and naturalistic characteristics. To provide patient-centered, effective healthcare, healthcare professionals need to inform their practice to consider and understand patients’ perceptions, thought processes, and implementation practices of CAM.

## Supplementary Information

**Additional file 1.** The participants’ positive perceptions of complementary and alternative medicine (CAM). A table in landscape that presents exemplifying quotes related to positive perceptions of CAM.

**Additional file 2.** The participants’ neutral perceptions of complementary and alternative medicine (CAM). A table in landscape that presents exemplifying quotes related to neutral perceptions of CAM.

## Data Availability

The datasets generated and/or analyzed during the current study are not publicly available due to participant privacy but are available in a redacted form from the corresponding author on reasonable request.

## Abbreviations

CAM: Complementary and Alternative Medicine
NCCIH: National Center of Complementary and Integrative Health
WHO: World Health Organization
UHM: University of Hawaiʻi at Mānoa
SBM: Social-Behavior Model
HCP: Health Care Provider
