# Supplementary material for: A qualitative investigation of the perceptions of complementary and alternative medicine among adults in Hawaiʻi
Source: BMC Complement Med Ther. 2022 May 7;22:128. doi: 10.1186/s12906-022-03603-3 (PMC9080192; doi:10.1186/s12906-022-03603-3)
Supplement: Supplementary file 1 — Additional file 1. The participants’ positive perceptions of complementary and alternative medicine (CAM). A table in landscape that presents exemplifying quotes related to positive perceptions of CAM. [file 12906_2022_3603_MOESM1_ESM.docx]

| **Additional file 1** The participants’ positive perceptions of complementary and alternative medicine (CAM) | | |
| --- | --- | --- |
| Positive Perception (n) | Definition | Exemplifying Quotations* |
| CAM is effective (8) | The perceived effectiveness of CAM due to obtaining desirable results. | “I notice that [essential oils] reduces my stress.” (P1) |
|  |  | “Alternative medicine has helped me solve issues and revert issues versus just masking symptoms.” (P4) |
|  |  | “I know that the herbal supplement works. It works well!”* (P5) |
|  |  | “The herbs mixtures for the common cold and symptoms have been very efficient. I was given [Traditional Chinese Medicine] to heal my sore throat. It worked very well and better than western medicine.” (P6) |
|  |  | “So I smoked a lot of weed and it works [for back pain].” (P8) |
|  |  | “My migraine spray seems to help a lot more [than conventional medicine].” (P9) |
|  |  | “The probiotic has helped me a lot getting a little bit more regular.” (P14) |
|  |  | “The muscle and fascia release [from massage therapy] allows me to be more functional, every day.” (P17) |
|  |  | “I had some muscle tightness in the glutes and I-T band which resulted in shin splints and the massage took care of that.” (P19) |
| CAM is better than conventional medicine (7) | The perceived notion that CAM is the preferred medical approach. | “I was given some TCM to heal my sore throat. It worked very well and better than western medicine.” (P6) |
|  |  | “I think [CAM] is a better alternative to heal the problem.”* (P10) |
|  |  | “I think [CAM] is a little bit more non-invasive, in a way. I mean, herbs are still potent, but my condition is that I have endometriosis and the only cure, or treatment, that they are offering is [conventional medicine]. I don’t particularly like to take [that medicine] because I feel like it’s messing with my body.* (P3) |
|  |  | “I like putting natural stuff in my body before I go to something that was made in a lab. I don’t know what kind of exactly biological process it could do to my body.”* (P5) |
|  |  | “And if there are other more, “natural” types of things to do, I’d lean towards that. Over... popping pills and stuff.” (P7) |
|  |  | “While taking the Western medicine I worry, “What about my kidney? What about my other stuff?” because Western medicine is like concentrated stuff from different kinds of chemicals rather than natural stuff.”* (P13) |
|  |  | “I didn’t like the side effects [of conventional medicine]. Also, I feel that I don’t have the same side effects that I've been experiencing with [conventional medicine].”* (P3) |
|  |  | “The benefits of CAM therapies seems to out-weigh, or lack any negative effect on my health.” (P4) |
|  |  | “You don't have that weird chemical hangover.” (P8) |
|  |  | “I don’t have really any side-effects.” (P12) |
|  |  | “[CAM] didn’t give me any side effects.” (20) |
|  |  | “I’m not a fan of conventional medicine, I would rather do CAM because it has helped me more than conventional medicine in my lifetime… it tends to treat the symptoms, not the root cause and that can cause more problems than solve. The benefits of CAM therapies seems to out-weigh, or lack any negative effect on my health.”* (P4) |
| CAM fosters well-being (5) | The perception of satisfaction after the CAM use related to feeling good or enjoying the treatment. | “Well I enjoy chiropractic, a lot. I do have pains all the time… And, I’ve never taken a pain killer before and I don’t think there is a need for it because I just enjoy the sensation of it [chiropractic] and the alleviation I get afterwards.”* (P7) |
|  |  | “Yoga makes me feel better.” (P9) |
|  |  | “Even though sometimes [TCM] tastes really bad – but then I feel like I'm better, I'm really feeling better.” (P13) |
|  |  | “I am very satisfied with [chiropractic] sessions because I feel like the chiropractor I have does a good job on making sure that my body is aligned, listens to all my needs, can pinpoint aches and things that I need to work on.”* (P17) |
|  |  | “I’m just satisfied because I’m doing natural stuff. I don’t want to put a bunch of drugs in my body because drugs have serious side effects. I feel like once you start doing drugs it’s kinda addicting.”* (P20) |
|  | Treat, not treatment | “[CAM] is not so much for therapy and more like maintenance of my health.”* (P4) |
|  |  | “I’ve used CAM therapies but not as a therapy.”* (P15) |
| *Quotations edited to add context and for grammatical purposes. P, participant number | | |

Legend: This table highlights the positive perceptions identified from participants. The three major themes leading to positive perceptions are the effectiveness of CAM, the belief that CAM is better than conventional medicine, and that CAM fosters well-being. Each theme has a definition to help code and organize the transcripts. The exemplifying quotations are the statements of participants that were identified to belong in the theme of positive perceptions.
