# Supplementary material for: A qualitative investigation of the perceptions of complementary and alternative medicine among adults in Hawaiʻi
Source: BMC Complement Med Ther. 2022 May 7;22:128. doi: 10.1186/s12906-022-03603-3 (PMC9080192; doi:10.1186/s12906-022-03603-3)
Supplement: Supplementary file 2 — Additional file 2. The participants’ neutral perceptions of complementary and alternative medicine (CAM). A table in landscape that presents exemplifying quotes related to neutral perceptions of CAM. [file 12906_2022_3603_MOESM2_ESM.docx]

| **Additional file 2** The participants’ neutral perceptions of complementary and alternative medicine (CAM) | | |
| --- | --- | --- |
| Neutral Perception (n) | Definition | Exemplifying Quotations* |
| Safety of CAM (14) | The perception that CAM is not likely to be harmful. | “I think there is safe practices but you have to be very cautious who you are gathering your information from.” (P1) |
|  |  | “I feel like it’s kind of a personalized thing. It could be safe and sometimes maybe, in certain situations, it may not be.” (P2) |
|  |  | “I think it depends on everyone’s condition and also the practitioner of the CAM depending on their knowledge.” (P3) |
|  |  | “It really depends on the specialist providing the CAM therapies… for example, acupuncture. You are having needles stuck into your body. You have to make sure the professional you seek the service from knows what they are doing because It can definitely adversely affect you if it’s not done correctly.” (P4) |
|  |  | “Well, I feel like for a healthy person it is, but then if you have health issues you might want to discuss that with your PCP.” (P5) |
|  |  | “I’d like the person know what they are doing for different types of traditional Chinese medicines and of course Chiropractics” (P7) |
|  |  | “I think maybe there is some drastic things that some people do… everything could be unsafe, conventional medicine too.” (P9) |
|  |  | “I think there is that risk of potential overdose or drug-herb interaction, drug-botanical interaction.” (P10) |
|  |  | “I guess it would be deemed unsafe if you do yoga and then you do it wrong or if you overdose on something.” (P11) |
|  |  | “It depends on who is using it. It definitely needs some education. Along with the use, which is probably why, it is not a conventional medicine.” (P12) |
|  |  | “I think it depends on how you’re using it.” (P14) |
|  |  | “It depends because sometimes when you mix herbs with other medications, some things could get complicated.” (P16) |
|  |  | “I don’t think it’s always safe... I was hurt more by physical therapists than I was helped”. (P17) |
|  |  | “Well, some is safe. Massage from a licensed practitioner. Some is not. There was saint johns’ wart that interfered with birth control products and others... Some is definitely unsafe, a lot is untested.” (P19) |
| CAM is natural (4) | The perception that CAM comes from nature and not created or manipulated by man. | “[CAM] is natural. I’m not putting any chemicals in my body. I’m not doing anything that my body is not meant to do.”* (P4) |
|  |  | “Alternative medicine for me is, what you put in your body, that's not like, synthetic. Ginger, chicken soup... that’s what I think of alternative medicine. Like, more healthy, natural ways of healing.” (P8) |
|  |  | “I guess there is a large perception of natural being somewhat better and because it’s coming from the earth so people are like, “Wow! This is how it’s supposed to be.” (P14) |
|  |  | “[CAM] comes from a plant. Not anything where a person is deliberately altering the chemicals of a substance to chemically change a product to do what they think it will do. It has to come exactly from nature with no alterations.”* (P20) |
| *Quotations edited to add context and for grammatical purposes. P, participant number | | |

Legend: This table highlights the neutral perceptions identified from participants. The two major themes leading to neutral perceptions are the natural characteristics of CAM and its safety. Each theme has a definition to help code and organize the transcripts. The exemplifying quotations are the statements of participants that were identified to belong in the theme of neutral perceptions.
